# Preeclampsia and Fetal Growth Restriction as Risk Factors of Future Maternal Cardiovascular Disease—A Review

**DOI:** 10.3390/jcm11206048

**Published:** 2022-10-13

**Authors:** Sylwia Sławek-Szmyt, Katarzyna Kawka-Paciorkowska, Aleksandra Ciepłucha, Maciej Lesiak, Mariola Ropacka-Lesiak

**Affiliations:** 1 1st Department of Cardiology, Poznan University of Medical Sciences, 61-848 Poznan, Poland; 2 Department of Perinatology and Gynecology, Poznan University of Medical Sciences, 60-535 Poznan, Poland

**Keywords:** cardiovascular disease, pregnancy complications, fetal growth restriction, preeclampsia, maternal morbidity

## Abstract

Cardiovascular diseases (CVDs) remain the leading cause of death in women worldwide. Although traditional risk factors increase later-life CVD, pregnancy-associated complications additionally influence future CVD risk in women. Adverse pregnancy outcomes, including preeclampsia and fetal growth restriction (FGR), are interrelated disorders caused by placental dysfunction, maternal cardiovascular maladaptation to pregnancy, and maternal abnormalities such as endothelial dysfunction, inflammation, hypercoagulability, and vasospasm. The pathophysiologic pathways of some pregnancy complications and CVDs might be linked. This review aimed to highlight the associations between specific adverse pregnancy outcomes and future CVD and emphasize the importance of considering pregnancy history in assessing a woman’s CVD risk. Moreover, we wanted to underline the role of maternal cardiovascular maladaptation in the development of specific pregnancy complications such as FGR.

## 1. Introduction

Cardiovascular diseases (CVDs) are the leading cause of women’s mortality globally, accounting for approximately one of every three female deaths [1]. The population-adjusted risk of CVDs-related death is significantly higher for women compared to men, 21% versus 15%, respectively [2]. Despite the significant decline in CVDs-related death in the last few decades, the mortality for women has decreased much slower than for men [3]. The underlying risk factors are frequently present many years before the clinical presentation of CVDs. Moreover, there has been growing evidence that women with a history of certain pregnancy complications are at increased risk of developing CVDs in the future [4,5]. These adverse pregnancy outcomes (APOs) include fetal growth disorders, gestational hypertension, or preeclampsia (See Table 1) [6]. 

Pregnancy acts as a maternal stress test, and the development of obstetric complications plays a potential role in a woman’s susceptibility to future CVDs. The etiologic pathways of pregnancy complications and CVDs might also be linked (e.g., metabolic syndrome, vascular dysfunction, or inflammation) [7]. The importance of these associations has been raised by the current guidelines, which now recommend a pregnancy history as a part of the routine evaluation of cardiovascular risk in women [8,9].

**Table 1:** Definitions of adverse pregnancy outcomes (APOs).

Type of APO	Definition
Gestational hypertension	De novo hypertension that develops after 20 weeks of pregnancy: - systolic blood pressure equal to or higher than 140 mmHg AND/OR - diastolic pressure equal to or higher than 90 mmHg on two separate occasions in a patient who was previously normotensive without proteinuria or other end-organ involvement [6].
Preeclampsia	De novo hypertension that develops after 20 weeks of pregnancy AND: 1. Proteinuria (≥300 mg/24-h of 0.3 g/g by urine protein: creatinine ratio or +1 by urine dipstick), OR 2. In the absence of proteinuria: - serum creatinine ≥ 90 µmol/L - alkaline or aspartate transaminase > 40 IU/L - platelet count < 150 000/µL - neurological complications (including altered mental status, blindness, stroke, severe headaches, clonus, and persistent visual scotomata) - uteroplacental dysfunction (including fetal growth disorder, abnormal umbilical artery Doppler waveform analysis or stillbirth) [10,11].
Fetal growth restriction (a) Early-onset (diagnosed before 32 weeks of gestation):	1. Fetal abdominal circumference below the 3rd percentile for gestational age OR 2. Estimated fetal weight below the 3rd percentile for gestational age, OR 3. The absence of end-diastolic flow of the umbilical artery on Doppler, AND - estimated fetal weight, or waist circumference below the 10th percentile for gestational age, AND - the pulsatility index of the uterine, and/or umbilical arteries above the 95th percentile for gestational age [12].
(b) Late-onset (diagnosed at or after 32 weeks of gestation):	1. Fetal abdominal circumference below the 3rd percentile for gestational age, OR 2. Estimated fetal weight below the 3rd percentile for gestational age, AND the combination of at least two of the following parameters: - estimated fetal weight or fetal abdominal circumference below the 10th percentile for gestational age, - the reduction in more than two quartiles in the growth curve - the cerebroplacental association below the 5th for gestational age - the pulsatility index of the umbilical artery above the 95th percentile for gestational age [12].
Gestational diabetes mellitus	One or more of the following criteria met at any time of pregnancy: - fasting plasma glucose 5.1–6.9 mmol/L (92–125 mg/dL) - 1-h plasma glucose ≥ 10.0 mmol/L (180 mg/dL) following a 75 g oral glucose load [13].

It is increasingly apparent that the effects of the maternal cardiovascular system maladaptation changes the predisposition to CVDs development after pregnancy. We aimed to systematically evaluate and quantify the evidence on the relationship between specific APOs’ and maternal risk of future cardiovascular disease. However, other factors, such as diabetes, renal impairment, or other dysmetabolic conditions will not be included in the analysis.

## 2. Preeclampsia

Preeclampsia is a pregnancy-specific disorder with an estimated incidence of 2–8% of all gestations associated with high maternal, fetal, and neonatal morbidity and mortality worldwide [14,15,16]. A detailed definition of preeclampsia is provided in Table 1. There is growing evidence of long-term cardiovascular sequelae in women who had preeclampsia during pregnancy [17,18].

### 2.1. Preeclampsia and Maternal Cardiovascular Risk

Several studies have shown the relationship between preeclampsia and future maternal CVDs [19,20,21,22,23]. The CHAMPS (Cardiovascular Health After Maternal Placental Syndromes) study indicated a more than a 2-fold increased risk of CVD (defined as hospital admission or revascularization for coronary artery, cerebrovascular, or peripheral artery disease at least 90 days after the delivery discharge date) in women affected by preeclampsia with absent traditional CVD risk factors (HR: 2.1; 95% CI: 1.8–2.4), and approximately 12-fold increased risk of CVD in women with a history of preeclampsia and metabolic syndrome (hazard ratio [HR]: 11.7; 95% confidence interval [CI]: 4.9–28.3) as compared to women with neither [19]. Apart from the pregnancy-specific factors and age, other risk factors are shared by preeclampsia and CVDs, but a direct causative relationship has not yet been determined. Lin et al., in a study performed on a Taiwanese cohort, demonstrated an increased risk of major adverse cardiovascular events including myocardial infarction, cardiogenic shock, heart failure, stroke, malignant dysrhythmia, or any other condition requiring percutaneous cardiac intervention, coronary artery bypass, an implantable cardiac defibrillator, or thrombolysis within three years of a preeclamptic pregnancy (HR: 12.6; 95% CI: 2.4–66.3) [20]. Kestenbaum et al., showed more than a 3-fold increase in cardiovascular events (hospitalizations due to MI, stroke, or percutaneous coronary artery interventions) (HR: 3.3; 95% CI: 1.7–6.5) and a higher number of thromboembolic events (HR: 2.3; 95% CI: 1.3 to 4.2) among women with previous severe preeclampsia during a mean follow-up of approximately eight years [22]. Furthermore, a Norwegian population-based cohort study with a median 13-year follow-up found an increased risk of CVD-related death defined as coronary artery disease, disease of the pulmonary circulation, or other diseases affecting the heart in women with a history of preeclampsia during pregnancy (RR: 1.65, 95% CI: 1.01–2.70), but the risk of CVD-related death was markedly higher in women with preeclampsia and preterm delivery (RR: 8.12, 95% CI: 4.31–15.33) as compared to women with a history of uncomplicated pregnancy [23]. The results of the British CALIBER (Cardiovascular Research using Linked Bespoke Studies and Electronic Health Records) study were similar, along with the reported overall first-time cardiovascular event incidence of 2.77% in the first nine years after a delivery complicated by preeclampsia in contrast to a 1.4% rate in women after an uncomplicated pregnancy [24].

Recent metanalysis reported that even after adjusting for potential confounders including age, body mass index, and diabetes mellitus, preeclampsia was related to increased risk of heart failure (RR: 1.6, 95% CI: 0.73–3.5), stroke (RR: 1.18; 95% CI, 0.95–1.46), coronary artery disease (RR: 1.46; 95% CI: 0.95–2.25), and death because of coronary artery disease (RR: 2.10; 95% CI, 1.25–3.51) or cardiovascular disease (RR: 2.21; 95% CI, 1.83–2.66), more than ten years after a pregnancy affected by preeclampsia. However, the increase in the risk for heart failure, stroke, and CVD-related death was even higher during the first decade after a pregnancy complicated with preeclampsia [25].

Notably, another large cohort study indicated an elevated risk of CVD-related death in women with a history of preeclampsia (HR: 2.14; 95% CI: 1.29–3.57), with a further significant risk acceleration if preeclampsia occurred before 34 weeks of gestation (HR: 9.54; 95% CI: 4.5–20.26) [26]. Moreover, Riise et al., reported a further increase in the CVD risk defined by coronary artery disease, after the recurrence of preeclampsia (HR 2.20; 95% CI: 0.91–5.32 in recurrent preeclampsia and HR 1.95; 95% CI: 1.31–2.91 for a single pre-eclampsia pregnancy), compared with uncomplicated pregnancies. When preeclampsia was combined with FGR or preterm birth, the risk was markedly higher (HR 4.66; 95% CI: 2.31–9.37 in recurrent preeclampsia as in comparison to one episode of preeclampsia; HR 2.81; 95% CI: 1.70–4.61) [27]. Other studies also support these results [RR 2.40; 95% CI: 2.15–2.68] [28,29,30]. Details are presented in Table 2. A higher frequency of heart failure (HR 4.2; 95% CI: 2.9–6.1) and cerebrovascular disease (HR 3.0; 95% CI: 1.70–4.61) among women with recurrent preeclampsia compared to women with unaffected pregnancy has also been reported [31].

### 2.2. Potential Mechanisms Linking Preeclampsia and Development of Future Maternal Cardiovascular Diseases

A few potential explanations for the association between preeclampsia and CVD are discussed. It has been proposed that preeclampsia may contribute as a predictor of cardiovascular events through distinct pathways [25]. On the other hand, the link between future CVD and preeclampsia may in part be due to shared risk factors between these entities. An unfavorable cardiovascular risk profile characterized by dyslipidemia, insulin resistance, diabetes, obesity, or endothelial dysfunction, heightened inflammatory responses, and hypercoagulable states frequent in preeclamptic women may result in an increased risk of CVD [14,25,40]. Another theory, which may be related to the above-mentioned, includes permanent vascular changes with excessive endothelial dysfunction that mediate the risk for future CVD [41].

Preeclampsia is characterized by pathological remodeling of the placental vessels, which is considered the main cause of uteroplacental ischemia. Spiral arteries do not undergo a physiological transformation and retain thick walls with a narrow lumen. The unsuccessful remodeling of spiral arteries results in high-velocity maternal blood flow at the intervillous space (approximately of 1–2 m/s) with a high spurt destroying the villi and forming thrombus-lined echogenic cystic lesions which can also be released to maternal circulation [42]. Furthermore, remodeling failure results in a repeated cycle of placental ischemia/reperfusion and leads to endothelial dysfunction, increases in the formation of reactive oxygen species (ROS) and the release of inflammatory cytokines and antiangiogenic factors, and maternal immune cell imbalance [43]. ROS decreases the bioavailability of proangiogenic factors such as nitric oxide which can result in impaired vasodilation, and angiogenesis, and increases the bioavailability of antiangiogenic factors such as soluble fms-like tyrosine kinase 1 (sFlt-1) soluble Endoglin (sEng) [44]. sFlt-1 is linked with defective angiogenesis and endothelial dysfunction by binding vascular endothelial growth factor and placental growth factor, while sEng which is a cell-surface co-receptor of transforming growth factor β (TGF β) initiates the proliferation and migration of endothelial cells [42]. It is believed that these antiangiogenic biomarkers strongly contribute to endothelial damage during the preeclamptic pregnancy, but do not remain significantly elevated after the delivery. It is hypothesized that the vascular damage sustained during the preeclamptic pregnancy persists and contributes to its own cascade in the CVD development in these patients [41]. Moreover, ROS disrupts maternal endothelial function by releasing cell-free fetal DNA and extracellular vesicles such as exomes into the maternal circulation [42].

Endothelial dysfunction is associated with inflammation and in consequence atherosclerosis [43]. The lipid deposition in the walls of the uterine spiral arteries resembles the early stages of atherosclerosis [45]. During early atherogenesis, low-density lipoprotein delivers cholesterol to the activated macrophages, which scavenge lipids. In an inflammatory milieu, the cholesterol cannot be recycled back into the circulation to the liver instead, it is trapped in the macrophages due to impaired reverse cholesterol transport in inflammation [46]. Acute atherosis in spiral arteries is represented by subendothelial lipid-filled foam cells enriched by CD68-positive macrophages, arterial wall fibrinoid necrosis, and perivascular lymphocytic infiltration [46,47]. It is also postulated that T regulatory cells (Tregs) protect against the development of atherosclerosis by downregulating effector T cell responses, despite their main role in maternal immunoregulation. However, the Tregs differentiation is stimulated by TGF β. As the above-mentioned TGF β is modulated by sEng, so it seems that high concentrations of sEng could locally inhibit the generation of Tregs cells and thereby promote acute atherosis [46].

The novel hypothesis links cellular fetal microchimerism (cFMC) with acute atherosis and the future development of CVD [48]. cFMC arises when cells of fetal origin are released into maternal blood and tissues during pregnancy [48]. These cells are known to possess stem cell-like properties, with the potential to differentiate into endothelial cells, smooth muscle cells, or leukocytes, and may persist in maternal circulation for decades [49]. Recent studies reported that cFMCs are more frequent in pregnancies complicated by preeclampsia or severe FGR than in healthy pregnancies [50,51]. It is hypothesized that in the dysfunctional placenta, fetal cells transfer more freely into the maternal bloodstream and induce a maternal anti-fetal immune response towards the fetal cells expressing foreign HLA surface peptides. If fetal cells persist in the circulation or are engrafted in maternal endothelial cells, they could induce further inflammation, especially in vessel walls, and initiate the development of inflammatory arterial lesions, particularly as acute atherosis [48].

Another possible mechanism involved in endothelial dysfunction seen in both atherosclerosis and preeclampsia is abnormal endothelial to mesenchymal transition (EndMT) [43]. EndMT is a normal, complex, dynamic, and reversible process during pregnancy in which epithelial cells lose polarity and adhesiveness, change to a mesenchymal phenotype, and acquire increased mobility [52]. Abnormal EndMT is observed in preeclampsia and is frequent in atherosclerotic lesions and plays a role in plaque progression and calcification [43].

It is hypothesized that in preeclamptic women, the endothelial integrity is not fully restored and remains more sensitized to stress-related or inflammatory stimuli as observed in atherosclerosis [43]. It was recently reported that women with placental malperfusion lesions had an adverse cardiovascular profile comprised of microvascular rarefaction with abnormalities in circulating endothelial and antiangiogenic factors, higher blood pressure, and more atherogenic lipids years after delivery [53]. Drost et al., showed that women with a history of preeclampsia have higher levels of SE-selectin and pregnancy-associated plasma protein A(PAPPA) compared to women with healthy pregnancies a decade after PE, after adjustment for traditional CVD risk factors [54]. SE-selectin is a marker of endothelial dysfunction, while PAPPA is a metalloproteinase associated with the presence of vulnerable atherosclerotic plaques and myocardial infarction [54,55]. Metalloproteinases may be the link between placental alterations in pregnancy and CVD in later life, supporting the hypothesis that vascular alterations in preeclampsia lead to persistent vascular damage and early development of atherosclerosis [54]. It is also postulated that comorbidities (e.g., gestational diabetes) may worsen the clinical course of preeclampsia by sharing similar placental vascular alterations that synergistically increase the risk of future CVD [56,57].

It is also hypothesized that several common genetic or epigenetic mechanisms may predispose to both acute atherosis and atherosclerosis and future CVDs [58]. It was found that women with a polymorphism of the regulator of the G protein signaling (RGS2) gene are at a higher risk of both preeclampsia and acute atherosis [59]. Reduced expression of RGS2 has also been linked to arterial hypertension [58].

Furthermore, subclinical markers of CVD, such as coronary artery calcium score (CACS), are also significantly higher in women with a previous pregnancy complicated by preeclampsia even after adjustment for age, blood pressure, and body mass index [22,23,60,61,62]. Moreover, 47% of women with prior preeclampsia had coronary atherosclerotic plaques on coronary computed tomography angiography and 4.3% had significant stenosis [63]. Formerly preeclamptic women develop coronary artery calcifications on average five years earlier from the age of 45 years onwards than women with prior normotensive pregnancy [64]. It was hypothesized that the body may not recover from the changes in the vascular and metabolic systems associated with preeclampsia and may demonstrate in later life with future cardiovascular events [64]. Similarly, previous studies demonstrated an increase in carotid intima-media thickness, a marker of subclinical atherosclerosis augmentation index in women with previous preeclampsia compared to age- and parity-matched controls [65].

Recent studies have shown that extracellular vesicles (EVs) are essential mediators in preeclampsia-related maternal CVDs [66,67]. EVs are membrane-bound particles consisting of bioactive proteins, lipids, DNA, mRNA, and microRNA (miR) that participate in cell-to-cell communication [66]. Placenta-derived EVs interact with the maternal immune system resulting in vascular inflammation and endothelial injury [66]. Several miRNAs in the placenta or blood of women with preeclampsia have been reported to be upregulated or downregulated compared with healthy pregnant women [67]. In particular, has-miRNA-134 overexpression is hypothesized to be involved in the inhibition of trophoblast cell infiltration by targeting integrin beta-1. has-miRNA-134 has also been associated with atherosclerosis, particularly acute myocardial infarction [67]. has-miRNA-23a-3p overexpression has been demonstrated to be involved in the pathophysiology of myocardial infarction and heart failure [68]. has-miRNA-23a-3p upregulation has been reported in the pathophysiology of myocardial infarction and heart failure and vascular calcification [68]. The up-regulation of has-miRNA-499a-5p has been linked with hypertension, preeclampsia, and FGR [69].

Physiological heart hypertrophy, which occurs during pregnancy in response to volume overload and hormonal stimuli, enables the heart to fulfill its function without significant long-term detrimental effects on cardiac function [66,70]. Higher vulnerability of ischemia-reperfusion injury during pregnancy complicated by preeclampsia is associated with increased ROS generation and decreased threshold for triggering the mitochondrial permeability transition pore opening. Pregnancy also has an impact on the number and content of EVs affecting the function of the heart. Placenta-derived EVs may impact ischemia-related injury due to a higher generation of ROS and activation of apoptosis during pregnancy [40]. Recently, Powell et al., demonstrated that EVs from preeclamptic women contribute to arterial tone regulation. They documented that ex vivo exposure of isolated mouse mesenteric arteries to EVs purified from the plasma of pregnant women with preeclampsia led to constriction in response to intraluminal pressure and resistance to methacholine-stimulated relaxation [71]. Furthermore, it is also suggested that EVs-exosomes from preeclampsia contribute to the dissemination of endothelial damage by sequestering the free vascular endothelial growth factor (VEGF) in the maternal circulation [72,73,74,75,76,77,78].

Moreover, gravidas with preeclampsia have significantly lower levels of angiotensin II compared to normal pregnancy, and these women have exacerbated vascular responses to angiotensin II in later life which also contributes to impaired microvascular function [74,75]. Interestingly, the expression of neprilysin is significantly increased in preeclampsia [76]. Neprilysin is released into the maternal circulation bound to placenta-derived EVs [66]. Increased levels of neprilysin might contribute to the persistence of hypertension and cardiac remodeling after pregnancy. A few studies based on animal models found the important role of the upregulated endothelin-1-mediated signaling in reduced endothelium-dependent dilation in preeclampsia [41,77].

Another possible mechanism linking preeclampsia with future CVD is increased sympathetic activity [44]. It has been reported that maladaptive baroreceptor responses are associated with persistently reduced plasma volume in women after a pregnancy complicated with preeclampsia opposite to normal sympathetic activity in women with uncomplicated pregnancies who return to euvolemia [44,78].

The mechanisms underlying the development of heart failure in women with prior preeclampsia remain poorly understood. It was previously suggested that preeclampsia might be a part of a pathway that leads to impairments in cardiac function [79]. Miralles et al., reported that preeclampsia might have long-term effects on the maternal cardiovascular system independently of any predisposing conditions. They used the STOX1 (the first gene identified in human families with preeclampsia via positional cloning) mouse model of preeclampsia and showed the expression of this gene in the disruption of cytotrophoblast function, associated with a marked imbalance between nitrosative and oxidative stresses within the placenta. They observed left ventricular hypertrophy, fibrotic cardiomyocytes, kidney glomerulitis, and modified transcriptome profile of the endothelial cells in female mice with preeclampsia as compared to normotensive controls [80]. Transcriptomic analysis indicated the deregulation of 165 genes in the heart, mainly linked with cardiac hypertrophy, and of 1149 genes in purified endothelial cells, associated with inflammation and cellular stress [80].

Women with pregnancies affected by preeclampsia have evidence of biventricular diastolic dysfunction and impaired systolic strain despite preservation of global systolic function assessed by ejection fraction [81]. During a hypertensive pregnancy, a significant increase in ventricular mass and relative ventricular wall thickness is also observed, indicating that the disproportion between wall thickens and the increase in ventricular volume [82]. Additionally, preeclampsia might cause structural and functional vascular changes that, along with cardiac remodeling, may result in microcirculatory shortfall [83]. Several previous studies indicated that in 25% to 72% of these women, the above-mentioned cardiac adaptations persist and do not revert during the postpartum period, causing a higher vulnerability to develop cardiovascular disease in later life [84,85,86]. A recent metanalysis showed a higher left ventricular mass index in women with prior preeclampsia with a mean difference of 4.25 g/m2 (95% CI, 2.08–6.42) [87]. On the contrary, others reported either no increase in left ventricular mass or a reversion to a pre-pregnancy state [81,85,88,89,90].

Most studies demonstrated a reduction in global strain in all principal directions (radial, circumferential, longitudinal), whereas few studies with relatively low samples did not indicate a significant difference [81,85,91,92]. In comparison with the no preeclampsia population, they also demonstrated a lower E/A ratio and a higher E/e′ ratio with a mean difference of −0.08 (95% CI, −0.15, −0.01) and 0.84 (95% CI, 0.41, 1.27), respectively [87]. Therefore, subtle contractional dysfunction may already occur without loss of ejection fraction in formerly preeclamptic women, indicating global strain as a sensitive parameter for early detection of cardiac function abnormalities [85].

## 3. Fetal Growth Restriction

Fetal growth restriction (FGR) is a condition of placental etiology with characteristics of inappropriate maternal cardiovascular system adaptation during pregnancy. In this APO, the fetus does not reach its biological growth potential due to impaired placental function, which may result from a variety of factors [93]. It is estimated that FGR complicates up to 10% of pregnancies and is one of the leading causes of infant morbidity and mortality [93,94]. The etiology of FGR is complex and can be caused by maternal (hypertension, diabetes, cardiopulmonary disease, anemia, malnutrition, smoking, drug use), fetal causes (genetic factors, congenital malformations, fetal infection, multiple pregnancies), and placental causes (placental insufficiency, placental infarction, placental mosaicism) [94]. FGR has been classified based on gestational age at prenatal ultrasound diagnosis as early-onset—diagnosed before 32 weeks of gestation and late-onset—diagnosed at or after 32 weeks of gestation [93,95]. The recent international Delphi consensus proposed an algorithm-based definition of FGR, which combine information on multiple fetal growth indicators in a deterministic manner [12]. Details are presented in Table 1.

The etiology of FGR differs depending on whether we consider early or late-onset fetal growth restriction. In early-onset FGR, two possible mechanisms concerning placental abnormalities and maternal cardiovascular system adaptation explain why this disorder occurs [16]. Moreover, early-onset FGR is frequently associated with preeclampsia (60–70%). On the other hand, the late-onset FGR is suggested to unmask a preexisting sub-clinical maternal cardiac dysfunction [96].

### 3.1. Fetal Growth Restriction and Maternal Cardiovascular Risk

Previous studies indicated that women with a pregnancy complicated by FGR risk developing CVDs in later life. FGR, similarly to CVDs, is characterized by chronic inflammation and oxidative stress [97]. Conditions such as obesity, hyperlipidemia, insulin resistance, and hypertension are linked with FGR and CVDs [98,99]. Recently, Bijl et al., demonstrated that women with a pregnancy complicated by early-onset FGR had unfavorable short-term cardiometabolic profiles with frequent obesity and low high-density lipoprotein- cholesterol levels (<1.29 mmol/L) in comparison with a control group [100]. Borna et al., reported that delivery of a low-birth-weight child, a surrogate marker of FGR increases the risk of future coronary artery disease approximately 6.5- fold. This association is independent of cigarette smoking, hypertension, hyperlipidemia, diabetes mellitus, age, body mass index (BMI), and waist circumference [98]. On the other hand, the results of the HUNT (Nord-Trøndelag Health) study indicated that pregnancy complications including preterm gestational age, led to only minor improvements in 10-year CVD risk prediction for parous women, as estimated by changes in model discrimination and reclassification [7]. It was also reported that pregnancy complication history does not add to CVD risk stratification for women aged fifty years and older. However, at younger ages, pregnancy complication history is known to predict the development of conventional CVD risk factors and may improve clinical risk prediction before the age of fifty years [101].

### 3.2. Potential Mechanisms Linking Fetal Growth Restriction and Development of Future Maternal Cardiovascular Diseases

Although preeclampsia and FGR are different entities, they present as interrelated disorders and share similar pathogenesis of inadequate placentation, inflammation, and maternal vascular dysfunction [102]. It is postulated that FGR is a result of poor trophoblastic invasion leading to defective spiral artery remodeling and, consequently, increased maternal peripheral vascular resistance and cardiac afterload [46,103,104]. However, it is also hypothesized that altered cardiovascular function characterized by low maternal cardiac output (CO) and high systemic vascular resistance (SVR) can exist before pregnancy and cause inappropriate placental perfusion and hence trophoblast impairment [96,105,106]. The interplay between the SVR and CO causes a secondary increase in preload and greater contractility of the left ventricle (LV) and consequently increase in LV mass [5]. Recent studies demonstrated that women with pregnancies complicated with normotensive FGR have a persistent myocardial impairment [98,107]. Melchiorre et al., reported that two-thirds of women with pregnancies complicated by FGR had poorer diastolic reserve with impaired myocardial relaxation, and a third had overt diastolic chamber dysfunction despite a preserved geometry and ejection fraction as assessed 12 weeks after delivery [107]. Orabona et al., showed subclinical LV impairment in systodiastolic function with concentric remodeling and smaller LV volumes, a slight alteration in right ventricular systolic function, and left atrial strain, similarly to women with former preeclampsia [99]. During normal pregnancy, eccentric cardiac remodeling typically occurs. Currently, it is believed that concentric remodeling is strongly linked with cardiac fibrosis and has considerable prognostic value in the development of CVDs [108,109]. It was found that most patients with pregnancies complicated by FGR have abnormal LV strain values. The plausible underlying mechanism may be related to chronic inflammation and oxidative stress, which impair production and/or activation of intracellular mediators and result in myocardial stiffening and interstitial connective tissue deposition leading to overt diastolic dysfunction and relative ischemia [99]. However, due to the lack of longitudinal studies, it is not yet elucidated whether FGR causes permanent cardiovascular alterations or whether these women have preexisting impairments contributing to complicated pregnancies.

The reduced CO and Increased SVR ”re a’sociated with the activation of the renin-angiotensin-aldosterone system. Previous studies indicated dysregulated angiotensin-processing enzyme and neprilysin expression with the vasoconstrictor pathway predominance [110].

Vascular dysfunction signs in FGR include a small placenta, decidual arteriopathy, placental infarcts with loss of functional placental parenchyma, and abnormalities of the placental villous tree, involving distal villous hypoplasia [96]. Similarly to preeclampsia, repeated ischemia/reperfusion events are associated with increased levels of antiangiogenic sFlt-1 and suppressed secretion of proangiogenic placenta growth factor (PIGF) [111]. It is believed that the sFlt-1/PlGF ratio corresponds to the severity of placental vascular dysfunction [112]. Inadequate trophoblastic invasion with the persistence of smooth muscle in the vessel walls and vascular damage results in acute atherosis and fibrinoid necrosis of vessels [113,114]. Moreover, cFMCs are also detected in pregnancies complicated by severe FRF combined with impaired placental perfusion [48]. Moreover, patients with previous FGR had a more severe degree of endothelial dysfunction than those with previous preeclampsia [115].

Notably, it was proven that maternal genes might modulate fetal growth by altering the intrauterine environment and uterine blood flow or by directly inheriting genes that regulate fetal growth changing. An increased risk of coronary artery disease and FGR is associated with mutations in genes encoding G proteins, glucokinase, angiotensinogen, and coagulation factors [116,117,118,119].

Placenta-derived eVs are involved in maternal immunotolerance towards the fetal allograft, inflammation, and angiogenesis. The fraction of circulating placenta-derived eVs is reduced in FGR, likely because of the impaired placental trophoblast activity, and may act as fetal growth markers [120,121]. A few studies indicated upregulation of specific eVs containing miRNAs including miR-499a-5p, and miR-1-3p, miR-127-3p, and miR-519a, and downregulation of others, particularly miR-210, miR-518b as a common feature of placental insufficiencies including FGR [122,123,124]. Among dysregulated miRNAs in CVD, miR-499-5p and miR-127-3p are highly overexpressed in heart failure and myocardial infarction [125,126]. miR-210 is a well-known hypoxia miRNA, which is upregulated in normal cells exposed to hypoxia in various diseases. In CVD, miR-210 is thought to protect the cardiovascular system from potentially lethal injury by inhibiting cell apoptosis and promoting angiogenesis, thus potentially guiding to revascularization [127]. However, the potential links between these miRNAs on maternal cardiovascular system dysfunction remain unclear. Thus, more studies are required to investigate whether epigenetic factors and the expression of miRNAs are predictive in evaluating the association between FGR and lifespan risk of CVDs.

It is postulated that FGR following maternal chronic bacterial infections is strongly related to a higher risk for atherosclerosis. Moreover, atherosclerotic plaques contain bacterial DNA, and it seems reasonable that an infectious trigger underlies the development of acute atherosis as well [48]. Exposure of human primary trophoblast to bacterial lipopolysaccharide leads to tumor necrosis factor α (TNFα) upregulation and macrophage accumulation in these cells [43]. TNFα is a strong inflammatory cytokine that is also upregulated in atherosclerosis. It was also demonstrated that in patients with FGR, the inflammatory response is stronger and remains stronger over time when compared to women with uncomplicated pregnancies [128]. Thus, TNFα seems to be a key player in several shared pathologic mechanisms of both FGR and atherosclerosis [43].

## 4. Summary

Adverse pregnancy outcomes such as FGR and preeclampsia are strongly related to long-term maternal CVDs risk. These pregnancy complications likely share common pathophysiologic pathways and are related to similar predisposing factors in women. Nonetheless, the intermediary mechanisms responsible for this association have not been sufficiently elucidated. Since pregnancy occurs early in a woman’s life, typically before the onset of clinically evident CVDs, it serves as a unique opportunity to evaluate a woman’s later life CVDs risk and introduce meaningful risk-reduction strategies. Future studies should address the issue of a structured screening for CVDs and the impact of timely preventive intervention in improving cardiovascular health in women with pregnancies affected by APOs.

## Figures and Tables

**Table 2:** Selected published studies of preeclampsia and future risk of cardiovascular disease.

Study/First Author (Reference)	Design	Population Size	Follow-Up (Period, Years)	Outcome Measure	Risk of Outcome Measures HR (95% CI)
Ray [20]	Retrospective	1,030,000	-	CVD	2.1(1.8–2.4)
Lin [21]	Registry	1,132,064	>3 years	Any MACE MI Heart failure Stroke MACE-related death	12.6 (2.4–66.3) 13.0 (4.6–6.3) 8.3 (4.2–16.4) 14.5 (1.3–165.1) 2.3 (1.6–3.1)
Kestenbaum [22]	Retrospective	31,239	-	CV events Thromboembolic events	2.2 (1.3–3.6) (mild pre-eclampsia) and 3.3 (1.7–6.5) (severe pre-eclampsia) 2.3 (1.3–4.2) (severe pre-eclampsia)
Irgens [23]	Registry	626,272	0 to 25 years (median 13 years)	CVD-related death	1.65, (1.01–2.70)—with preeclampsia 8.12 (4.31–15.33) with preeclampsia and preterm birth
Mongraw-Chaffin [26]	Retrospective	14,403	Median 37 years	CVD-related death	2.14 (1.29–3.57) 9.54 (4.50–20.26) if onset of preeclampsia before 34 weeks’ gestation
Auger [32]	Registry	1,108,581	0–25.2 years (Median 15.5 years)	CAD HF Cerebrovascular disease	3.3 (2.1–5.2) 4.2 (2.9–6.1) 3.0 (2.3–4.1)—with recurrent preeclampsia
CALIBER [24]	Registry	1,300,000	-	CAD Stroke Heart failure, Hypertension CVD-related death	1.67 (1.54–1.81) 1.9 (1.53–2.35) 2.13 (1.64–2.76) 4.47 (4.32–4.62) 2.12 (1.49–2.99)
Wikstrom [33]	Registry	403,555	15 years	CAD	1.6 (1.3–2.0) with GHA 1.9 (1.6–2.2) with mild preeclampsia 2.8 (CI 2.2–3.7) with severe pre-eclampsia
Smith [34]	Registry	129,920	15–19 years	CAD-related death	1.7 (0.9–3.3)
Lykke [35]	Registry	782,287	14.6 years	CVD-related death	2.08 (1.63–2.64)
Kessous [36]	Registry	96,370	10 years	CVD: hospitalization for CAD, stroke, peripheral vascular disease, hyperlipidemia, angina, hypertension, atherosclerosis, MI, heart failure, pulmonary heart disease, cardiac arrest, cardiac catheterization, or cardiovascular stress test	1.7 (1.6–1.9)
Crillo [37]	Registry	14,062	40 years	CAD-related death	3.6 (1.04–12.19)
Hannaford [38]	Prospective	23,000	Not available	Hypertension CAD Angina Venous thromboembolism	2.35 (2.08–2.65) 1.65 (1.26–2.16) 1.53 (1.09–2.15) 1.62 (1.09–2.41)
McDonald [39]	Metanalysis	2,375,751	Not available	CVD-related death Stroke Peripheral artery disease	2.29 (1.73–3.04) 2.03 (1.54–2.67) 1.87 (0.94–3.73)
Wu [25]	Metanalysis	6,400,000	Not available	CAD CAD-related death Heart failure stroke CVD-related death	1.46 (0.95–2.25) 2.1 (1.25–3.51) 1.6 (0.73–3.5) 1.18 (0.95–1.46) 2.21 (1.83–2.66)

Abbreviations: CAD, coronary artery disease; CV, cerebrovascular; CVD, cardiovascular disease; HF, heart failure; MACE, major adverse cardiovascular event; MI, myocardial infarction.

## Data Availability

Not applicable.
